# Supplementary material for: Through a Horse’s Eyes: Investigating Cognitive Bias and Responses to Humans in Equine-Assisted Interventions
Source: Animals (Basel). 2025 Feb 19;15(4):607. doi: 10.3390/ani15040607 (PMC11851653; doi:10.3390/ani15040607)
Supplement: Supplementary file 1 [file animals-15-00607-s001.zip › animals-3394614-supplementary.pdf]

Supplementary Materials

Table S1. Impact of the riding site (sites 1, 2 and 3), the type of work (riding school lessons and equine assisted intervention + riding school lessons) and their interaction on the latencies to touch the horses in the approach-contact tests and the latencies to fit the halter in the halter test (N=30).

	F	Df	*p*
**ACT**[1]			
Site	0.66	2	0.53
Work	0.29	1	0.59
Site*work	1.20	2	0.32
**Halter**			
Site	2.86	2	0.08
Work	1.05	1	0.32
Site*work	6.30	2	**0.02**

[1]ACT: approach contact test; the Likelihood Ratio Test statistics (F), the denominator degrees of freedom (Df), and p values (LMM, **bold values** $p < 0.05$; *italic values* $0.05 < p < 0.08$) are shown.

Table S2. Median latency (in seconds) in the judgment bias test to go to the bucket for the positive (P) and negative (N) learnt locations and the three ambiguous locations: near positive (NP), middle (M), and near negative (NN); (N=30).

	Median	Q1-Q3[1]	Median EAI-RS[2]	Q1-Q3	Median RS[3]	Q1-Q3
P	9.5	8.25-15	9.13	7.94-13.12	9.88	8.31-15.69
NP	8	7-8.75	7	7-11.75	8	7-8.75
M	7.5	7-11.75	7	7-26.25	9	6.25-10.75
NN	19	8-52.75	9	8-19.5	38	8-108.5
N	180	127.5-180	180	138.75-180	180	140.5-180

[1]Q1-Q3: first and third quartiles; [2]EAI-RS: equine assisted intervention + riding school horses (N=16); [3]RS: riding school horses (N=14).

Table S3. Relationship between adjusted latency in the three ambiguous bucket locations near positive (NP), middle (M), and near negative (NN) in the judgment bias test and the total working time (in hours) per week, EAI working time (in hours) per week or EAI percentage of total working time (N=30).

	Total working time	Total EAI working time	Percentage of EAI working time
NP adjusted latency	r=0.24, $p = 0.20$	r=-0.09, $p = 0.65$	r=-0.19, $p = 0.29$
M adjusted latency	r=0.16, $p = 0.39$	r=-0.01, $p = 0.95$	r=-0.04, $p = 0.84$
NN adjusted latency	r=0.07, $p = 0.72$	r=-0.27, $p = 0.14$	r=60.31, $p = 0.09$

Spearman correlation tests, r: rho coefficients, p: p values.

Table S4. Relationship between human perception by horses measured as positive, indifferent, aggressive or fear reactions for each horse in the approach contact and the halter tests and judgment bias adjusted latency within the three ambiguous bucket locations: near positive (NP), middle (M), and near negative (NN); (N=30).

	F	Df	*p*
**Scores in ACT**[1]			
NP adjusted latency	2.70	3	*0.07*
M adjusted latency	0.12	3	0.95

NN adjusted latency	2.14	3	0.12
**Scores in the Halter test**			
NP adjusted latency	0.09	3	0.23
M adjusted latency	0.21	3	0.54
NN adjusted latency	0.53	3	0.41

[1]ACT: approach contact test; the Likelihood Ratio Test statistics (F), the denominator degrees of freedom (Df), and p values (LMM, **bold values** $p < 0.05$; *italic values* $0.05 < p < 0.08$) are shown.

Video S1: Horse go response towards a positive bucket location;
Video S2: Horse no-go response towards a near-negative bucket location.
